# CT chest of COVID-19 patients: what should a radiologist know?

**DOI:** 10.1186/s43055-020-00245-8

**Published:** 2020-07-07

**Authors:** Tamer F. Ali, Mohamed A. Tawab, Mona A ElHariri

**Affiliations:** 1 Department of Radiodiagnosis, Faculty of Medicine, Zagazig University, PO. BOX 184, Sharkia, Zagazig 44511 Egypt; 2 Department of Radiodiagnosis, Faculty of Medicine, Al-Azhar University, Cairo, Egypt

**Keywords:** COVID-19, GGO, CT chest, Pneumonia, Consolidation

## Abstract

**Background:**

The aim of current work is to review the CT findings of COVID-19 in a pictorial study to help the radiologists to be familiar to imaging findings of COVID-19.

**Main body:**

Coronavirus disease 2019 (COVID-19) is a pandemic highly infectious disease which is first reported in December 2019 in Wuhan, China, and then had its outbreak leads to a global public health emergency. Real-time reverse transcription polymerase chain reaction (RT-PCR) of viral nucleic acid is considered as the reference standard for COVID-19 diagnosis; however, recent studies showed the importance of CT chest in the diagnosis of COVID-19 with high sensitivity.

The CT hallmarks of COVID-19 were bilateral peripheral ground-glass opacities, consolidation with the advance of the disease more consolidation is there with linear opacities and crazy-paving pattern as well as halo and reverse halo sign.

**Conclusion:**

Early identification of COVID-19 cases is vital. The radiologist should be familiar with the possible findings. Further future studies with pathological correlation will help for more understanding of the imaging findings and its value in assessing of prognosis.

## Background

Coronavirus disease 2019 (COVID-19) is a pandemic highly infectious disease which is first reported in December 2019 in Wuhan, China, due to severe acute respiratory syndrome coronavirus 2 (SARS-CoV-2). On January 30, 2020, the World Health Organization (WHO) announced this outbreak as a global public health emergency and raised it to the very high risk on February 28, 2020 [1–7].

Understanding the clinical presentation of COVID-19 is of big importance; the clinical presentations are variable and can include fever as a major presentation; other symptoms include fatigue and cough, while some literature reported diarrhea and nausea to precede fever. Some cases can be asymptomatic, while elderly cases with comorbidities are more vulnerable for respiratory failure. While patients may have normal or low WBCs, RBCs and platelet count with prolonged activated thromboplastin time and increased CRP [5–8].

Real-time reverse transcription polymerase chain reaction (RT-PCR) of viral nucleic acid is considered as the reference standard for COVID-19 diagnosis [8–11]; however, recent studies showed the importance of CT chest in the diagnosis of COVID-19 with high sensitivity [11-20].

According to the official diagnosis and treatment protocol (6th edition) issued by the National Health Commission of China [21], CT examination is of great help in diagnosis, follow-up, and treatment evaluation.

COVID-19 can utilize angiotensin-converting enzyme-2 (ACE2) as the cell receptor [22], so affect pulmonary interstitial pathology the parenchymal abnormalities resulting in CT changes that can be variable depending of the severity and stage of the disease [22–24].

While peripheral posterior bilateral ground-glass opacities (GGO) with or without consolidation was the major hallmark of COVID-19, other variable CT findings were reported [25–31].

The aim of current work is to review the CT findings of COVID-19 in a pictorial study to help the radiologists to be familiar to imaging findings of COVID-19.

## Main text

### CT manifestations of COVID-19 (Figs. 1, 2, 3, 4, 5, 6, 7 and 8)

#### Pulmonary findings

##### Ground-glass opacity

GGO is defined as hazy lung areas of slightly increased attenuation without obscuring the bronchial and vascular margins; it can be caused by partial air displacement attributed to partial airspaces filling or interstitial thickening [32, 33]. It is considered the most common radiological abnormality seen in up to 98% in COVID-19 cases [9, 21–24].

**Fig. 1:**
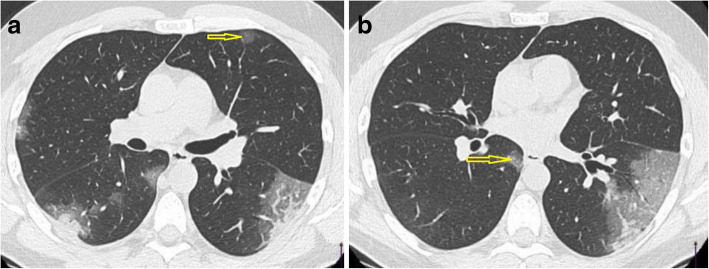
CT chest axial cuts of COVID-19 patient. A: ground glass opacity (anterior segment of left upper lobe, yellow arrow) and combination of GGO and consolidation in both lower lobe (peripheral, subpleural). B: ground glass opacity (medial segment of right lower lobe, yellow arrow) as well as GGO and consolidation of left lower lobe (peripheral, subpleural)

**Fig. 2:**
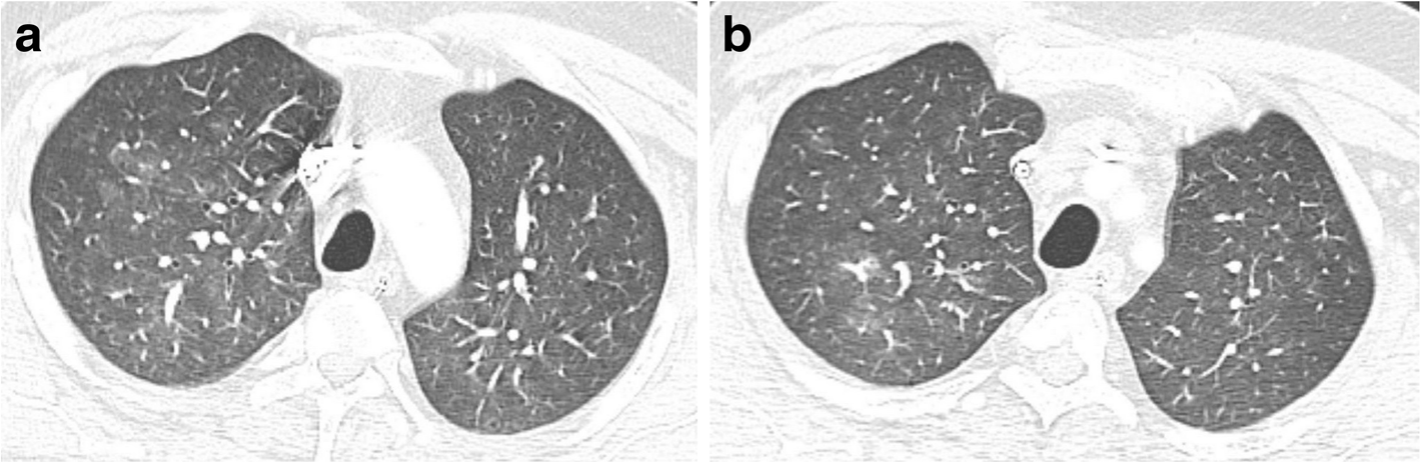
**a**, **b** CT- chest of COVID-19 patient shows widespread faint ground glass opacity upper lung lobe as well as reticular shadowing bilaterally

**Fig. 3:**
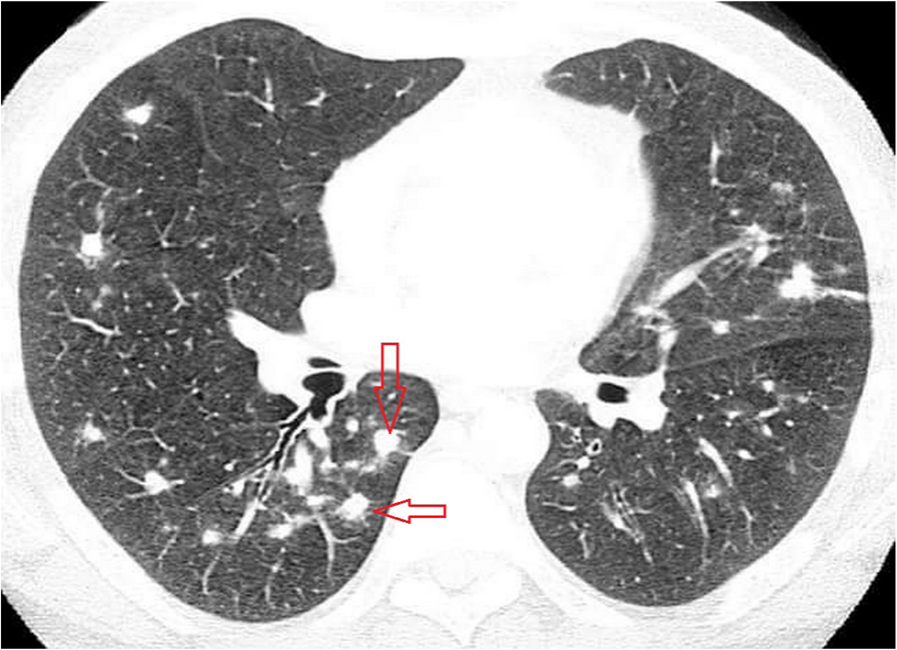
CT scan shows multiple irregular nodules in right middle and lower lobe, lingula and anterior segment of left lower lobe with solid nodules surrounded by a ground glass (red arrow)

**Fig. 4:**
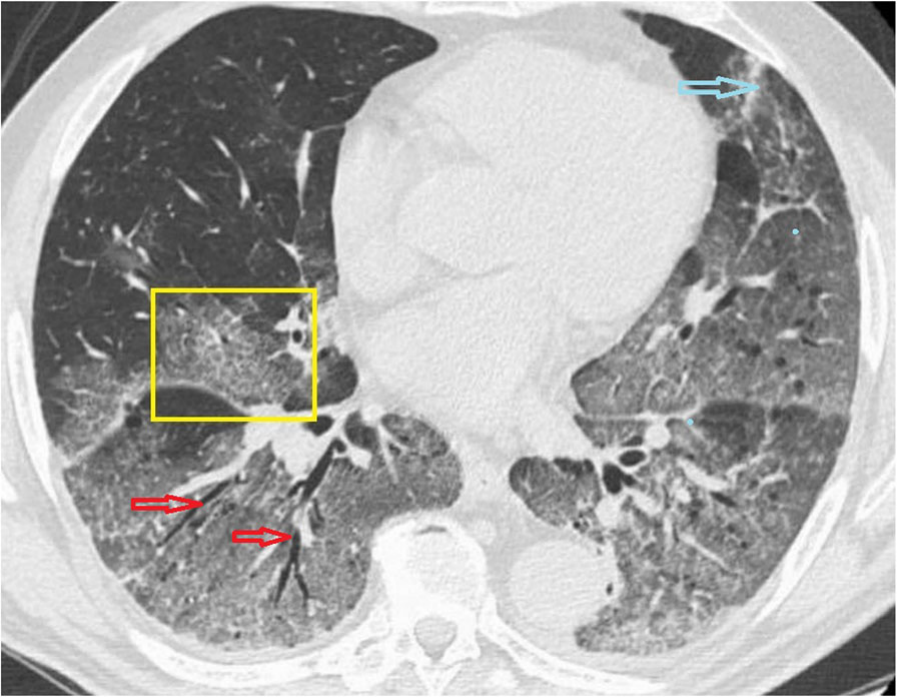
CT scan of COVID-19 patient shows reticular pattern superimposed on the background of GGO with crazy paving stones signs at anterior segment of right lower lobe (yellow box), bronchiolar dilatation (red arrows) at right lower lobe, fibrotic changes (blue arrow) are also noted

**Fig. 5:**
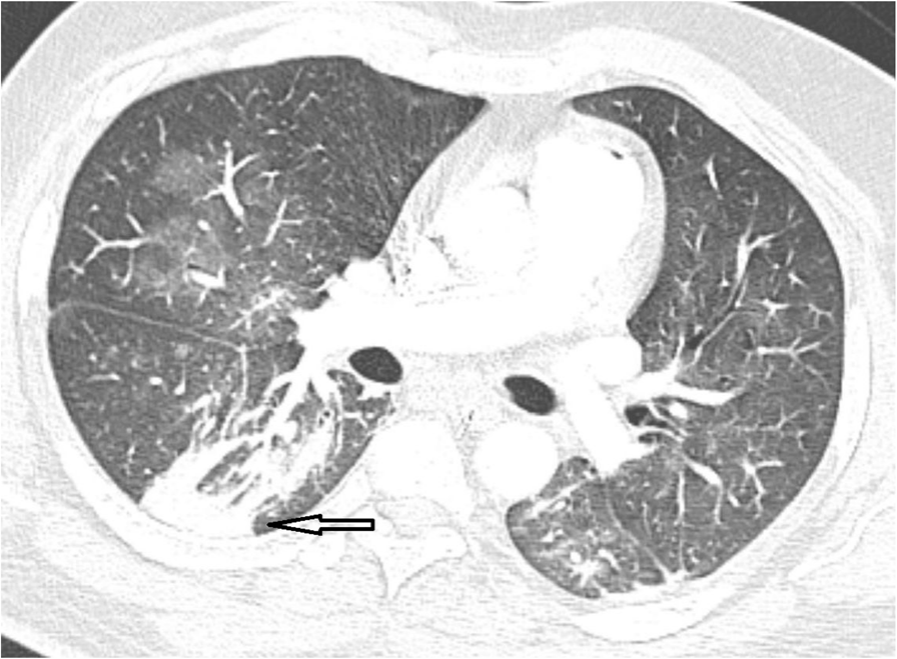
CT scan of COVID-19 patient shows Multiple areas of GGO as well as reticular pattern at anterior segment of upper lobes as well as superior segments of lower lobes

**Fig. 6:**
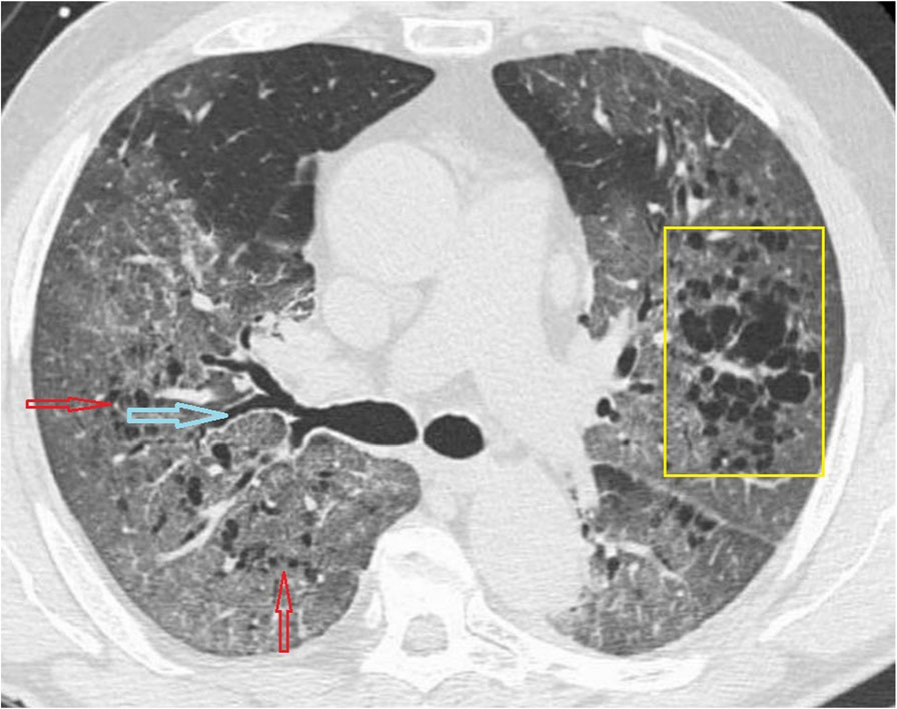
CT scan of COVID-19 patient shows reticular pattern superimposed on the background of GGO with air trapping at lower lobes (red arrow) as well as cystic changes at left lower lobe (yellow box). Bronchiolar irregularity at right lower lobe (blue arrow)

**Fig. 7:**
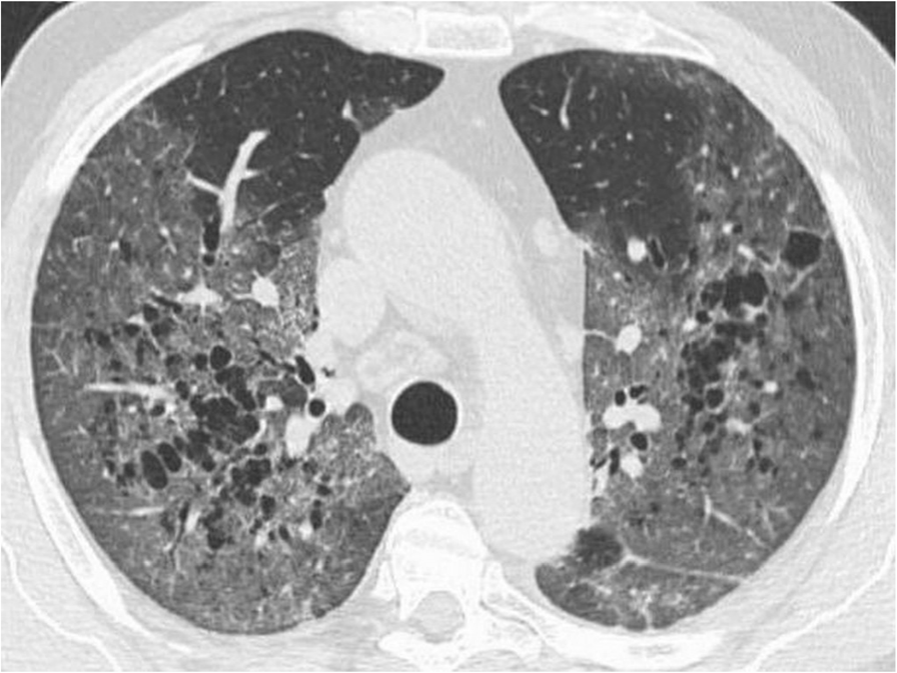
CT scan of COVID-19 patient shows reticular pattern superimposed on the background of GGO with nodular shadows and bilateral small cystic changes at upper lobes

**Fig. 8:**
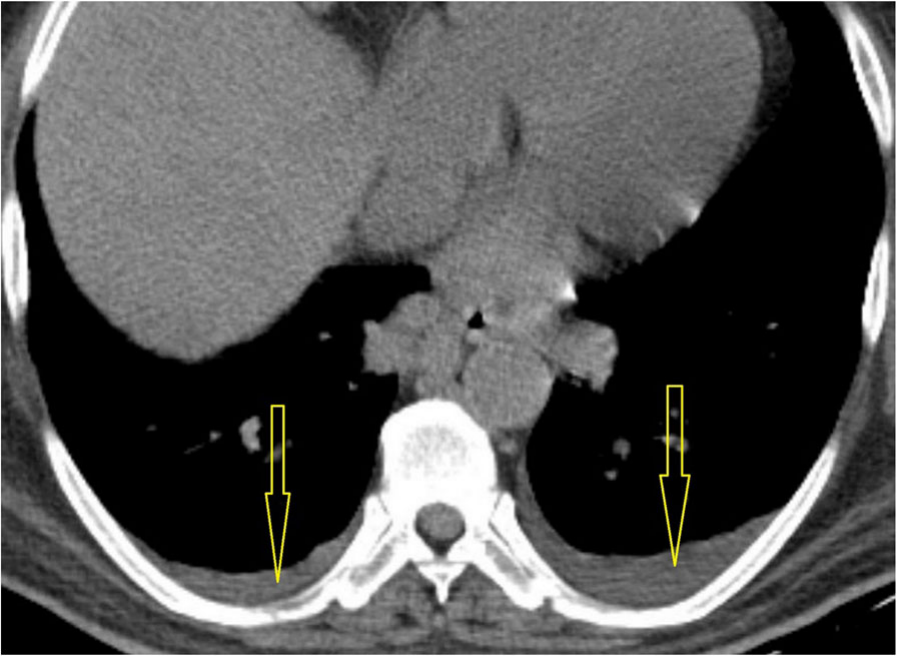
CT scan of COVID-19 patient shows minimal bilateral pleural effusion (yellow arrows)

In COVID-19, GGO is predominately peripheral and subpleural, either uni or bilateral [12–17]. In earlier study by Chung et al. [18], GGO was considered as earliest CT finding and detected in 57% of cases.

##### Consolidation

Consolidation is defined as increase in the pulmonary parenchymal attenuation with obscuration of the vascular and airway wall margins due to pathological replacement of alveolar air by fluids, cells, or tissues. It can be multifocal, patchy, or segmental with subpleural location or along bronchovascular bundles [32, 33].

Literature shows consolidation prevalence to be about 2–64% in COVID-19 cases [11, 19, 20]. The pathology of consolidation of COVID-19 can be attributed to alveolar cellular fibromyxoid exudates [34]. Consolidation can occur as a progress of the disease or co-existed with GGO [20, 23].

##### Reticular pattern

Reticular pattern refers to thickening of the pulmonary interstitial structures as interlobular septa and intralobular lines [32, 33]; with linear opacities seen at CT studies, it can be explained by the interstitial lymphocyte infiltration [34]. Reticular pattern was noted in many COVID-19 CT studies, up to 70.6% of cases [1] which increases with prolonged course of the disease [13, 20].

##### Crazy-paving pattern

The crazy-paving pattern is a linear pattern superimposed on a GGO background giving the appearance of irregular paving stones (crazy); it can be explained by alveolar edema and acute lung interstitial inflammation [32, 35]. It was reported in 5–36% COVID-19 patients [9, 11]. It can be a sign of disease progression [9, 24].

##### Air bronchogram

Air bronchogram refers to the pattern of air-filled bronchi (dark) on the background of opacified alveoli (grey/white) [32] and was noted in previous studies of CT chest for COVID-19 [9, 13, 27]; however, the hypoattenuation of bronchi can be attributed to gelatinous mucus and not air [36]. Thus, some suggested to call it bronchiolectasis [9].

##### Bronchus deformation

It is described as airway changes (bronchiectasis and thickening of bronchial wall thickening); it was reported in 10–20% of COVID-19 patients [1, 9, 11, 15, 22, 26]. It can be attributed to the inflammatory changes of bronchial wall leading to obstruction and subsequent destruction of bronchial wall with fibrosis and bronchiectasis change [9, 32].

##### Fibrosis

Lung fibrosis was reported at CT studies of COVID-19 cases in previous series [12] in about 17% of cases. It can be explained by the replacement of cells by scar tissue in the healing process of chronic pulmonary inflammation; thus, it is considered to be a good prognosis by some author [12], while others claimed that it is of poor prognosis as it proceeds to interstitial pulmonary fibrosis disease [28, 35].

##### Pathological air containing spaces

It is described as small air spaces within the lung; it can be due to pathological dilatation or air spaces or due to resorption of consolidation; some author described it as cavity [28], and other called it cystic changes [23] and cavity [32] or bubble sign [9].

##### Subpleural line

It is described as thin (1–3 mm thickness) curvilinear opacity that is parallel to the pleural surface and less than 1 cm deep to it [32]. It was described in about 20% of COVID-19 cases in some series and can be explained by pulmonary edema or fibrosis that takes place in COVD-19 [11, 20].

##### Vascular enlargement

Pulmonary vessel dilatation, peri- and intralesional, was reported in some series [9]; however, it is assumed to be rare [25], while other reported it in 82.4% of cases [1]; it can be explained by capillary wall damage in response to inflammatory process-related factors [9].

##### Pulmonary nodules

Nodule is described as a small (less than 3 cm) round, oval, or irregular shaped well or poorly defined opacity in the lung [32]. It was reported in 3–13 of COVID-19 CT cases and mostly was multifocal irregular and can have halo sign [1, 9, 12, 31, 37].

##### Halo sign and reversed halo sign

Halo sign is described as ground-glass opacity surrounding a pulmonary nodule or mass and represents hemorrhage which was typically described in angioinvasive fungal infection or hypervascular deposits and was attributed to perifocal hemorrhage [31, 32, 38]. In COVID-19, it was described in some report to be about 17.6% but pathology is not understood yet [1, 31]. On the other hand, reversed halo sign (atoll sign) is described as a central ground-glass opacity with denser surrounding crescentic or ring consolidation [32]; it was noted in COVID-19 cases in 3.9% of cases [1] and explained by the progression of disease so that consolidation surrounds GGO or lesion regression with its center being of low attenuation [9, 19, 27, 39].

#### Extrapulmonary findings

##### Pleural changes

Pleural thickening was reported in COVID-19 with higher incidence than effusion [23] with prevalence of up to 32% of COVID-19 cases in some series [19, 23], while pleural effusion was reported as a rare finding in COVID-19 cases with incidence of 2% and may be a bad prognostic sign [11, 23]; furthermore, some suggested that occurrence or pleural effusion and extensive tiny pulmonary nodules to be due to bacterial superinfection [29].

##### Mediastinal lymphadenopathy

It describes enlarged lymph nodes (> 1 cm short axis) [32] which may be assign of severe COVID-19 infection; it was reported in 0–8% of COVID-19 cases [1, 20, 23].

##### Pericardial effusion

Pericardial effusion was earlier reported as being rare (< 5%) finding in COVID-19 cases and can be explained by the severe inflammatory process; some reports described it with a higher incidence in critical COVID-19 cases than milder cases [11].

So, the CT findings in COVID-19 are variable. While mild cases can show bilateral ground-glass opacities and small areas of subsegmental consolidation with the advance of the disease, there can be bilateral multiple subsegmental and lobular consolidation. In advanced cases, CT can reveal heterogeneous widespread bilateral with air bronchogram and bronchial deformation as well as septal thickening and crazy-paving appearance.

## Conclusion

Isolation of COVID-19 patients is of critical importance to control its spread; thus, early identification of cases is vital. While GGO and consolidation are on top of COVID-19 CT chest findings, the radiologist should be familiar with other possible findings. Further future studies with pathological correlation will help for more understanding of the imaging findings and its value in assessing of prognosis.

## Data Availability

The data that support the findings of this study are available on request from the corresponding author.

## Abbreviations

ACE2: Angiotensin-converting enzyme-2
COVID-19: Coronavirus disease 2019
CRP: C-reactive protein
CT: Computed tomography
GGO: Ground-glass opacities
RBC: Red blood cells
RT-PCR: Real-time reverse transcription polymerase chain reaction
WBC: White blood cells
WHO: World Health Organization
